# Surgical Kidney

**Published:** 1913-11

**Authors:** A. L. Fowler

**Affiliations:** Atlanta, Ga.; Professor of Genito-Urinary Surgery in the Atlanta Medical College; Consulting Genito-Urinary Surgeon to the Grady (Municipal) Hospital; Genito-Urinary Surgeon to St. Joseph Infirmary; Member American Urological Association, Etc.


### Journal-Record of Medicine

Successor to Atlanta Medical and Surgical Journal, Established 1855
and Southern Medical Record, Established 1870

**OWNED BY THE ATLANTA MEDICAL JOURNAL COMPANY**

PUBLISHED MONTHLY

*Official Organ Fulton County Medical Society, State Examining Board, Presbyterian Hospital, Atlanta, Birmingham and Atlantic Railroad Surgeons' Association, Chattahoochee Valley Medical and Surgical Association, Etc.*

EDGAR BALLENGER, M. D., Editor
BERNARD WOLFF, M. D., Supervising Editor
A. W. STIRLING, M. D., C. M., D. P. H.; J. S. HURT, B. Ph., M.D.
GEO. M. NILES, M. D., W. J. LOVE, M. D., (Ala.); Associate Editors
E. W. ALLEN, Business Manager

**COLLABORATORS**

DR. W. F. WESTMORLAND, General Surgery
F. W. McRAE, M. D., Abdominal Surgery
H. F. HARRIS, M. D., Pathology and Bacteriology
E. B. BLOCK, M. D., Diseases of the Nervous System
MICHAEL HOKE, M. D., Orthopedic Surgery
CYRUS W. STRICKLER, M. D., Legal Medicine and Medical Legislation
E. C. DAVIS, A. B., M. D., Obstetrics
E. G. JONES, A. B., M. D., Gynecology
R. T. DORSEY, JR., B. S., M. D., Medicine
L. M. GAINES, A. B., M. D., Internal Medicine
GEO. C. MIZELL, M. D., Diseases of the Stomach and Intestines
L. B. CLARKE, M. D., Pediatrics
EDGAR PAULIN, M. D., Opsonic Medicine
THEODORE TOEPEL, M. D., Mechano Therapy
R. R. DALY, M. D., Medical Society
A. W. STIRLING, M. D., Etc., Diseases of the Eye, Ear, Nose and Throat
BERNARD WOLFF, M. D., Diseases of the Skin
E. G. BALLENGER, M. D., Diseases of the Genito-Urinary Organs

VOL. LX. ATLANTA, GA., NOVEMBER, 1913. NO. 8

### SURGICAL KIDNEY.

BY A. L. FOWLER, M. D.,
ATLANTA, GA.

Professor of Genito-Urinary Surgery in the Atlanta Medical College; Consulting Genito-Urinary Surgeon to the Grady (Municipal) Hospital; Genito-Urinary Surgeon to St. Joseph Infirmary; Member American Urological Association, Etc.

In suppurative pyelitis, synonymous with surgical kidney, the pelvis of one kidney may be attacked, or both may be involved.

The diseases, pyelitis, pyelo-nephritis, pyo-nephrosis, nephritic abscess and peri-nephritic abscess are very closely associated one with another, so that it is an event of rarity to find any single one of them existing singly.

By pyelitis we mean an inflammation of the kidney pelvis; pyelonephritis is the same thing, plus a nephritis; and pyonephrosis is pyelitis plus a nephritis, but with additional complication of enlargement of the pelvic cavity, the same being due to destruction of renal substances by abscess, or dilation from obstructions, such as stone in the kidney, or ureter, ureteral stricture, hypertrophied prostate, or urethral stricture. By peri-nephritic abscess we mean a collection of pus around, or about the kidney. Abscess of the kidney and suppurative nephritis, both comparatively rare, occur independently of the above group.

### ETIOLOGY.

Suppurative diseases of the kidney, whether they begin in the pelvis and by simple extension invade the parenchyma, or vice-versa, are always caused by micro-organisms.

The infection may reach the organ through the blood (haematogenous infection), or through the lymph (lymphogenous infection), or from neighboring structures (contigious infection), and, lastly, it may ascend by continuity of the mucosa from either the urethra, bladder, or ureter. The French school, which has done such exhaustive investigation in this connection (Albarran, particularly), conclude that the circulatory route is the more common.

The various pus producing organisms known to cause these suppurative conditions are: the colon bacillus (most common), staphylococcus, streptococcus, bacillus pyocyaneous, and the Proteus vulgaris. The gonococcus is occasionally, though not often, the micro-organism giving rise to suppuration in the kidney. The tubercle bacillus, though not strictly regarded as a pus producing organism, frequently attacks the kidney when, as a result of its lowered vitality, conditions are produced which favor the reception of pus producing organisms.

PREDISPOSING CAUSES.—Congestion or irritation may adapt the kidney favorably for the growth of organisms, and an inveterate cystitis back of a hypertrophied prostate, or tight stricture, may also act as a predisposing cause. The urine becomes dammed back upon the kidneys with resultant distension and infection of the pelves, via the ureters.

A renal calculus does not, of itself, excite a pyelitis, but the prolonged mechanical irritation eventually makes easy the entrance of germs. An abundance of calcium oxalate, or other crystals may act in the same way.

SURGICAL KIDNEY.

PATHOLOGY.—Early in the disease, only a simple catarrh of the mucosa of the pelvis, with hyperaemia of the superficial capillaries and increase of mucus, exists. But as the infectious process progresses the wall becomes thickened and is lined by a purulent exudate. If the infection be tubercular the process is accentuated, and active erosions become a feature.

The pelvis is observed to be more largely dilated when the infection is an ascending one but, on the other hand, the surface of the mucous membrane is smoother and thinner, and the capillary congestion is not so marked. While the amount of urine and pus is larger, the admixture is thinner than in the descending cases. Whenever there is obstruction due to tuberculosis or calculus, retention will occur within the pelvis in varying degrees. But it always occurs in a much greater degree if the obstruction has for its site the ureter proper.

When pus and urine become retained within the pelvis the process, by simple extension, usually invades the parenchyma with a resultant pyelo-nephritis or pyo-nephrosis.

SYMPTOMS AND COURSE.

Pyelitis may be either acute or chronic, and by reason of the disease usually being secondary to some other condition, the symptoms are frequently obscured by those of the primary disease.

The acute form resembles those of acute ureteritis, from which it is impossible to distinguish it during life. It begins with a chill and high fever, and is accompanied by a dry tongue, thirst, and great prostration. The urine contains pus, albumin, and bacteria, and which varies with the kidney involvent. It is seldom that the kidney region is tender in the acute form. When tenderness is persistently and prominently present on palpation it is more likely indicative of abscess formation.

In the acute form sometimes death follows very quickly, but very often the temperature subsides suddenly or gradually. On the other hand, the fever may become remittent in type, until the temperature finally becomes normal when the acute attack merges into the chronic form.

The transition from the acute to the chronic form is recognized as not infrequent, but it must be remembered that chronic pyelitis usually begins insiduously, and that both pyelitis and pyelo-nephritis may run their course without fever.

Chronic pyelitis may persist, without change, for several years and during this time the parenchyma remains uninvolved. In low grades of infection a pyelonephritis may run such a chronic course, and the destruction of the kidney substance be so gradual, that it will be several years before any subjective symptoms become noticeable.

When the disease exists alone, and not associated with calculus, tumor, tuberculosis, or much renal mobility, the symptoms are few. At times they may be entirely absent.

Occasionally there is slight frequency of urination, or there may be an indefinite feeling of fatigue in one or both loins. If a calculus or tumor be present as a complication, the pain will be more intense and radiating; further, there will probably be a varying haematuria in consequence. The intensity of the pyuria will depend on whether there is a cystitis or not: because if the bladder be uninvolved the pyuria is mild. After the bladder becomes involved (descending infection) the frequency of urination is increased. If tubercular, the number is even greater. Attacks of nausea, vomiting, retention, chills and fever are generally due to renal calculus, or movable kidney. The extension of the inflammation to the kidney substance is indicated by febrile attacks, so we should always be on our guard lest this extension occur.

EXAMINATION.

In palpating the kidney in pyelitis, a slight tenderness over the kidney region may be elicited, but its absence does not signify that the kidney is healthy. Enlargement of the organ is absent unless a complication is present, such as retention of urine and pus within the pelvis, or extension to the kidney substance. It occasionally happens that, with a great amount of pus formation the pus, by reason of its being blocked in the ureter, drains backward into the kidney pelvis when it becomes larger and more tender. While the obstruction persists the urine becomes free from pus, and is quite clear, but the retention is most likely to be attended by fever and kidney colic.

From the vague and unreliable character of the symptoms we are obliged to depend on the changes in the urine, or rather the single symptom of pus in the urine. But since other diseases of the bladder, prostate and urethra cause the urine to be mixed with pus, it becomes necessary to determine if the pus comes from the kidney, and if so, which kidney?

It is of value to the examiner to remember that every case of cystitis, except those caused by malignant tumors, tuberculosis, and calculus, improves under local treatment of the bladder. If no diminution of the pus takes place, it follows that the pus is coming from the kidney or its pelvis. A manoeuvre, sometimes of value, is to wash out the bladder thoroughly, leaving the catheter in place, and then after fifteen minutes the accumulated urine is drawn off. If the kidneys are healthy, but cystitis is present, a small amount of pus will appear admixed with the urine. On the other hand, if the bladder is healthy (descending infection into the bladder having not yet occurred) and a pyelitis exists, the urine will contain a comparatively large amount of pus.

The urine in the chronic cases usually shows a urine of low specific gravity, somewhat increased in amount, containing much pus, serum and nucleo-albumin, pelvic epithelia, a few to fair number of red blood cells and, may be, hyaline and granular casts.

When the pyelitis is due to tuberculosis, tubercle bacilli may be found in the urine (in some cases guinea-pig innoculation alone will disclose them); while if the result of stone, crystals may be found en masse in pus and mucus, or free, the specific gravity being higher.

### Diagnosis.

The differential diagnosis between cystitis and pyelitis is sometimes a most difficult problem, especially in acute cases. It sometimes happens that the patient is suffering from both together. Bladder tenesmus, increased frequency of urination, and pyuria, are common to both conditions.

Among the interesting points it is well to remember that in chronic cystitis, the daily amount of urine and urea, unless the patient has been given a large amount of water and diuretics, are always normal. The reaction, too, is generally alkaline, or if not, it shortly becomes so, save in those instances due to the colon or the tubercle bacillus. The amount of albumin does not exceed that caused by the pus and blood, and there is a muco-purulent sediment which coagulates quickly. In simple cystitis the amount of albumin is seldom more than one sixth. If a case of cystitis does not clear up on lavage, pyelitis, posterior urethritis, and prostatitis should be considered and excluded.

Fever is seldom caused by a pure cystitis unless the urine is foul and intensely alkaline, so that fever with pus in acid urine is, in all probability, due to pyelitis.

In cystitis, the microscope will disclose pavement epithelia from the upper layers of the bladder, and cuboidal epithelia from its middle layers. If the cystitis be intense there will be columnar epithelia from the deep layers; if very intense there will be found connective tissue shreds. But there will be no epithelia from the renal pelvis or tubules. There is neither renal pain, no tenderness on pressure over the kidney.

The urine in pyelitis is acid, seldom alkaline, contains a larger amount of albumin than the pus can account for (one-sixth or more), and the pus sinks less slowly and is far more profuse than in cystitis; and fever, with pus in acid urine, is in all probability due to pyelitis.

If there is pain or tenderness in one or both loins and, of even greater importance, if the temperature is raised to 100° F., the symptoms are due to pyelitis.

These cases should always be carefully palpated which may disclose a tender, swollen kidney, and the X-ray may show a renal calculus.

In chronic pyelitis there is polyuria, the sediment is more diffuse, and it does not coagulate quite so quickly. The importance of the colon bacillus, whose recognition is not difficult, is not generally appreciated.

A golden rule of Legueu and Kidd is; "Never be content with a diagnosis of spontaneous cystitis until pyelitis has been excluded".

The only certain test is cystoscopy and ureteric catheterization, which should seldom be carried out in acute cases. In the acute cases the surgeon had better remain satisfied with a provisional diagnosis of pyelitis if he can not verify his suspicions by microscopic findings. It should be remembered that the number of small signs and symptoms cause the disease to be entirely missed clinically, and which is revealed many, many times at post-mortem.

In catarrhal pyelitis (we are equally justified in speaking of catarrhal pyelitis, just as we are in speaking of catarrhal nephritis and catarrhal cystitis), the microscope discloses the same features as in catarrhal nephritis, except that pelvic epithelia, instead of kidney epithelia, are found. As the disease, in many cases, is due to an abundance of urinary salts, there will be present the crystals of the particular salt occasioning the disease.

In acute suppurative pyelitis, the diagnosis can be made from the cuboidal and irregular pelvic epithelia, which are not only abundant, but may be found in groups. These large epithelia are irregular, angular or pear-shaped. Other features are: red blood corpuscles in moderate or even large numbers, and a profuse number of pus corpuscles. Further, connective tissue shreds are abundant and without them a diagnosis of acute suppurative pyelitis should not be made.

### Prognosis.

If the cause can be removed, the patient generally recovers. This may require a great length of time, or it may never take place. In the latter instance the inflammatory process extends upwards to the kidney substance, with a resultant pyelo-nephritis or a pyo-nephrosis.

### Treatment.

The treatment of the acute form is usually that of the expectant plan. Narcotics for the pain, application of ice to the lumbar region antipyretics' for the temperature, urotropin to antisepticize the urine, a bland diet, copious draughts of some good spring water, and a cholagogue are the orthodox treatment.

If, under this treatment, the active symptoms do not subside, and the symptoms become threatening, such as high fever and chills and the involved kidney having been determined, a nephrotomy sometimes is attended with brilliant results.

The treatment of the chronic form is pretty much on the same lines. Some require confinement and rest, while others may be allowed to be about. A bland and unstimulating diet, plenty of good spring water, rest, baths, urotropin and benzoate of soda, of each grs. 7½, sometimes cure cases of mild type. The chances are small that the lesions will heal.

Dilatation of the pelvis with an albuminate of silver solution, while the patient is in the Trendelinburg position, has given some good results. Nitrate of silver instilled into the kidney pelvis, in cases due to gonococci and colon bacilli infection, has been loudly praised by Casper and others. Irrigations of the pelvis are contraindicated in the presence of pyelonephritis, tuberculosis, and renal calculus.

Lavage of the pelvis should be tried before proceeding to operation.

### Operative Treatment.

#### One Kidney Alone Diseased.

In one-sided pyelitis, not too far advanced, nephrotomy gives good results. If the kidney is riddled with small abscesses the operator should not hesitate to do nephrectomy at once, provided the opposite kidney is capable of sustaining the patient, as disclosed by functionating test and ureteral catheterization.

If nephrotomy is done the pelvis should be irrigated with a 1-1000 silver nitrate solution, the wound being kept open and drained.

#### Both Kidneys Diseased.

Here, nephrectomy can not be considered. In double-sided pyelitis it is better to do a nephrotomy on one side, and then after draining the kidney for a sufficient time, do a nephrotomy on the opposite kidney.

If too much destruction has not occurred the evacuation of the pus may relieve the septic condition, and the sound parts of the kidney may be able to carry on their eliminative function sufficiently to sustain life.

It has been well pointed out by Morton that in feeble old men, who are suffering from stricture and enlarged prostate, with both kidneys diseased, no operation can be undertaken which holds out much prospect of recovery.

The author lays no claims to originality in this paper, and has gathered his data from the writings of some of the foremost urologists in this country and abroad.

Bibliography: Legueu, Kidd, Heitzmann, Zuckerhandl and Albarran.

929 Candler Building, Atlanta, Ga.

